# A preoperative inflammatory score-based nomogram predicts overall survival after curative hepatectomy for hepatocellular carcinoma

**DOI:** 10.1007/s12672-025-03406-1

**Published:** 2025-08-31

**Authors:** Xiaojian He, Xu Peng, Jianliang Duan, Zongyang Li, Yuexiang Niu, Yahui Liu

**Affiliations:** Department of Hepatobiliary and Pancreatic Surgery, General Surgery Center, The First Hospital of Jilin University, Changchun, Jilin China

**Keywords:** Hepatocellular carcinoma, Hepatectomy, Inflammation indices, Nomogram, Overall survival, Prediction model

## Abstract

**Objective:**

This study incorporated preoperative inflammatory scores to develop and validate a nomogram to predict overall survival in patients with hepatocellular carcinoma following curative resection.

**Methods:**

The study included 402 postoperative hepatocellular carcinoma patients, divided into training (n = 281) and test (n = 121) cohorts. Variables were analyzed using Cox proportional hazards model. The nomogram’s performance was assessed using receiver operating characteristic curves, calibration curves, and decision curve analysis.

**Results:**

Multivariable Cox proportional hazards model analysis identified neutrophil-to-lymphocyte ratio–lymphocyte-to-monocyte ratio score (HR = 4.19, 95% CI 2.47–7.12), microvascular invasion (HR = 4.93, 95% CI 2.74–8.85), and total tumor volume (HR = 1.67, 95% CI 1.03–2.68) as independent prognostic factors (*P* < 0.05). The nomogram exhibited excellent discriminatory ability, with area under the curve values for 12-, 36-, and 60-month overall survival in the test cohort measuring 0.941, 0.810, and 0.881. Calibration curves verified a high degree of consistency, with a Brier score of 0.054, 0.120, and 0.102, between the predicted and observed survival probabilities in the test cohort. Decision curve analysis confirmed clinical utility across a wide threshold probability range (0.15–0.70).

**Conclusion:**

The nomogram integrating neutrophil-to-lymphocyte ratio–lymphocyte-to-monocyte ratio score, microvascular invasion, and total tumor volume effectively identifies high-risk hepatocellular carcinoma patients with shorter overall survival. This tool provides clinicians with new evidence for risk-stratified interventions.

**Supplementary Information:**

The online version contains supplementary material available at 10.1007/s12672-025-03406-1.

## Introduction

In 2022, approximately 865,269 individuals worldwide were diagnosed with liver cancer, with hepatocellular carcinoma (HCC) representing the most common histological subtype. HCC ranks as the third leading cause of cancer-related mortality, with a 5-year relative survival rate of approximately 18% [1–3]. China accounts for the highest burden of liver cancer, with 42.5% of global cases originating from the region according to GLOBOCAN 2022 data [3]. Recent global statistics highlight significantly higher incidence and mortality rates among individuals in China compared to the global average, with annual incidence and mortality rates of 15 and 8.6 per 100,000, respectively. HCC constitutes approximately 80% of primary liver cancer in China. Hepatectomy remains the cornerstone of treatment for HCC and is the preferred modality for early-stage disease [4]. Despite advancements in surgical guidelines and techniques over the past decades, post-resection overall survival (OS) for HCC patients remains suboptimal. A critical limitation is the absence of robust prognostic models, leading to inadequate post-resection interventions and surveillance, which compromise overall survival [5].

The pivotal role of inflammation in predicting cancer prognosis has been increasingly recognized [6]. Inflammatory biomarkers, including the neutrophil-to-lymphocyte ratio (NLR) and lymphocyte-to-monocyte ratio (LMR), derived from neutrophil, lymphocyte, and monocyte counts, have emerged as independent prognostic factors in multiple malignancies [7–10]. Elevated inflammatory scores have been widely used to predict tumor progression and recurrence [11–14]. Although NLR and LMR have been studied in various cancers, their association with post-resection OS in HCC remains incompletely understood. As cost-effective and accessible biomarkers, these indices hold promise for prognostic stratification in HCC. This study evaluates the correlation between preoperative inflammatory scores and OS in HCC patients after hepatectomy, integrating clinical variables to develop a nomogram for aiding clinical decision-making.

## Methods

### Study population

We retrospectively collected data from 402 HCC patients who underwent curative hepatectomy at the Department of Hepatobiliary and Pancreatic Surgery, General Surgery Center, the First Hospital of Jilin University, between March 2020 and March 2022. The inclusion criteria were as follows:

(1) Initial HCC resection surgery with pathologically confirmed hepatocellular carcinoma, (2) No preoperative interventional therapy, chemotherapy, targeted therapy, radiotherapy, or ablation therapy, (3) Absence of adjacent organ invasion, intrahepatic metastasis, lymph node metastasis, or distant metastasis, (4) Preoperative liver function classified as Child–Pugh grade A or B according to the Child–Pugh scoring system.

The exclusion criteria were: (1) Post-resection pathology indicating combined hepatocellular-cholangiocarcinoma, cholangiocarcinoma, metastatic liver cancer, or other hepatic malignancies, (2) Positive resection margins confirmed by post-resection pathology, (3) Post-resection adjuvant interventional therapy, chemotherapy, targeted therapy, radiotherapy, or ablation therapy, (4) Combined surgical resection with radiofrequency ablation, microwave ablation, or 5-fluorouracil implantation, (5) Concurrent diagnosis of HCC with other malignancies or life-threatening comorbidities, (6) Incomplete follow-up data or patients lost to follow-up.

Based on these criteria, a total of 402 patients were retrospectively enrolled and randomly assigned to a training cohort (n = 281) and a test cohort (n = 121) in a 7**:**3 ratio.

### Laboratory examinations and histopathology

Before the surgery, we recorded clinical parameters including age, gender, hepatitis B surface antigen (HBsAg), NLR, LMR, alpha-fetoprotein (AFP) levels, albumin (ALB), alanine aminotransferase (ALT), aspartate aminotransferase (AST), indirect bilirubin (IBIL), white blood cell count (WBC), neutrophil count (NEU), lymphocyte count (LYM), monocyte count (MONO), platelet count (PLT), prothrombin time (PT), surgical approach, blood loss, tumor number, total tumor volume (TTV), microvascular invasion (MVI). The study additionally examined the Barcelona Clinic Liver Cancer (BCLC) staging system.

NLR and LMR were calculated from indices obtained from the most recent preoperative blood test. The NLR was determined as the neutrophil count divided by the lymphocyte count, while the LMR was determined as the lymphocyte count divided by the monocyte count. TTV was calculated as the sum of the volume of each tumor [(4/3) × 3.14 × (a × b × c)] cm^3^, where a, b, and c represent the three semi-axes (in cm) of each tumor [15–18].

MVI is defined as the presence of micro-metastatic HCC emboli within hepatic vasculature, including both the hepatic venous and portal venous systems. According to the classification system established by Feng et al., MVI is categorized into four classes based on two critical criteria: endothelial invasion status of tumor cells and the total number of involved vessels. Specifically, these classes are defined as M0 (no MVI), M1 (non-invasive tumor emboli with fewer than five involved vessels), M2 (either invasive emboli with fewer than five involved vessels or non-invasive emboli with five or more involved vessels), and M3 (invasive emboli with five or more involved vessels) [19].

### Survival data

Post-resection follow-up assessments were conducted every 3–6 months, including telephone interviews and outpatient clinic visits. The follow-up period was completed on December 29, 2024. The primary endpoint was OS, defined as the time from hepatectomy to HCC-related death.

### Statistical analyses

Statistical analyses were conducted using IBM SPSS Statistics 25.0 and R software (version 4.3.1). Categorical variables were compared using Chi-square or Fisher's exact test and continuous variables with the Mann–Whitney U-test. Kaplan–Meier survival curve and log-rank test were used to evaluated OS. Least absolute shrinkage and selection operator (LASSO) and multivariable Cox proportional hazards model analysis were employed to identify factors influencing post-resection OS in HCC patients undergoing hepatectomy. A nomogram for OS after hepatectomy was developed using R software (version 4.3.1). The Hosmer–Lemeshow goodness-of-fit test was applied to evaluate the calibration of the nomogram in both training and test cohorts, accompanied by visualization of calibration curves. The predictive value of the nomogram for OS in post-resection HCC patients from the training and test cohorts was evaluated through receiver operating characteristic (ROC) curve analysis. The accuracy and effectiveness of the predictive model were evaluated using decision curve analysis (DCA). A two-sided *P* < 0.05 was regarded as an indication of statistical significance.

## Results

### Patient characteristics

**Fig. 1:**
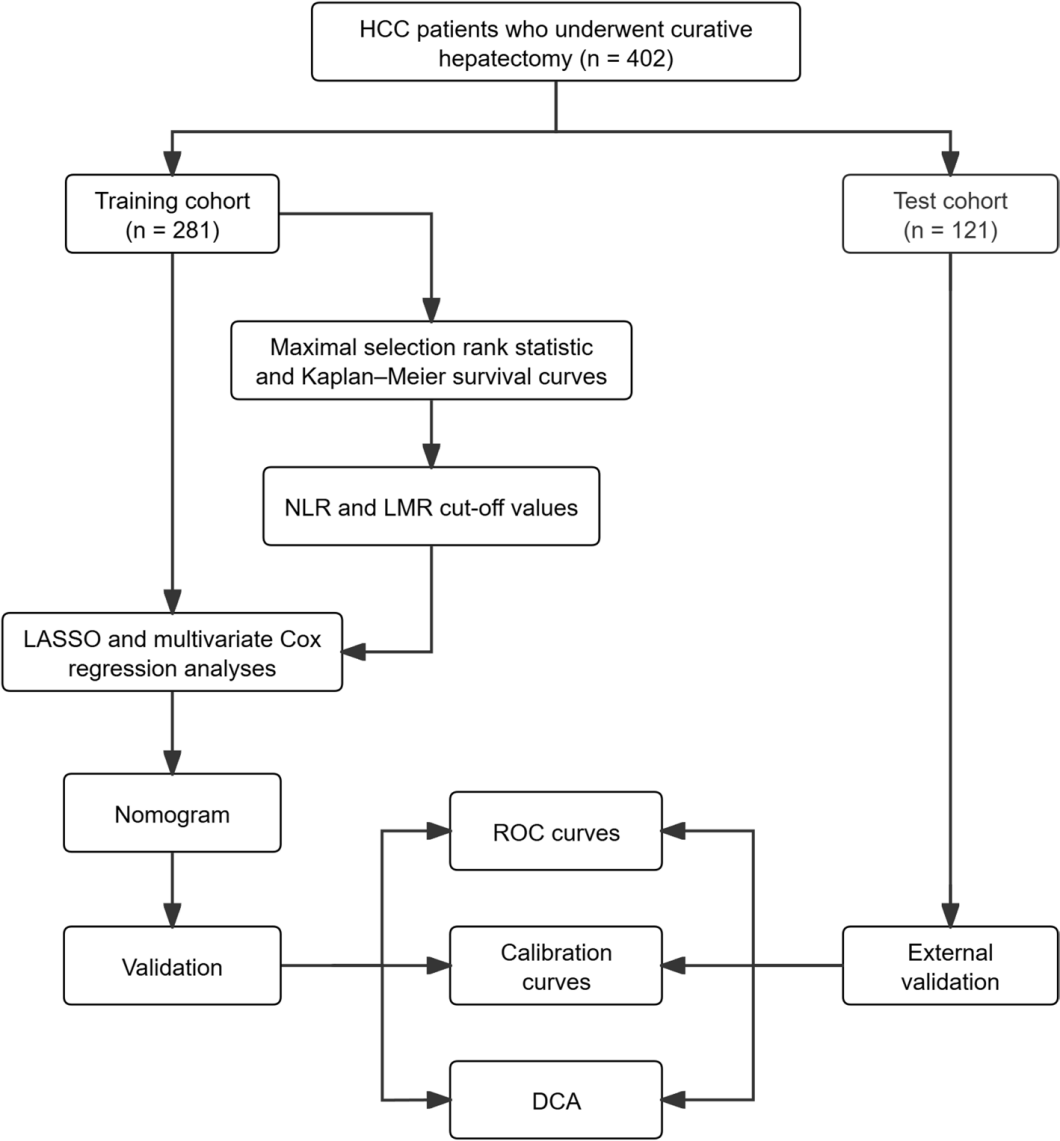
Flow chart of the study

This study included 402 HCC patients who underwent curative hepatectomy. Demographics were as follows: 311 males (77.4%) and 91 females (22.6%). Hepatitis B surface antigen (HBsAg) positivity was observed in 290 patients (72.1%), and 338 (81.4%) had solitary intrahepatic tumors. MVI staging revealed M0 in 203 cases (50.5%), M1 in 145 (36.1%), and M2 in 54 (13.4%). Child–Pugh scores were Class A in 368 patients (91.5%) and Class B in 34 (8.5%). BCLC staging included Stage 0 in 39 (9.7%), Stage A in 287 (71.4%), Stage B in 69 (17.2%), and Stage C in 7 (1.7%). Patients were randomly allocated to a training cohort (n = 281) and a test cohort (n = 121) in a 7**:**3 ratio. There were no significant baseline clinical differences between groups (*P* > 0.05) (Table 1, Fig. 1).

**Table 1:** Baseline characteristics of training and test cohorts

Variables	Total (*n* = 402)	Training (*n* = 281)	Test (*n* = 121)	*P* value
Gender, n (%)				
Male	311 (77.4)	213 (75.8)	98 (81.0)	0.254
Female	91 (22.6)	68 (24.2)	23 (19.0)	
Age, years, median (IQR)	56.0 (51.0–64.0)	56.0 (51.0–63.0)	56.0 (51.0–64.0)	0.632
HBsAg, n (%)				
Negative	112 (27.9)	73 (26.0)	39 (32.2)	0.200
Positive	290 (72.1)	208 (74.0)	82 (67.8)	
PIVKA-II, ng/mL	124.9 (33.7–637.1)	147.7 (36.5–757.3)	90.4 (27.9–558.3)	0.887
AFP, ng/mL	20.6 (3.9–450.1)	18.4 (3.9–531.2)	24.0 (4.0–386.3)	0.956
ALB, g/L	38.4 (35.4–40.7)	38.4 (35.4–40.7)	38.3 (35.2–40.5)	0.792
ALT, U/L	27.8 (18.9–41.6)	27.7 (18.9–41.4)	27.7 (18.9–41.5)	0.345
AST, U/L	29.2 (20.8–44.5)	28.8 (21–43.5)	29.9 (20.3–44.9)	0.689
IBIL, μmol/L	10.9 (8.0–14.2)	10.9 (8.1–14.9)	11 (8.0–13.5)	0.369
WBC, × 10⁹/L	5.6 (4.2–6.9)	5.6 (4.4–7.1)	5.5 (4.1–6.5)	0.225
NEU, × 10⁹/L	2.9 (1.8–4.2)	2.9 (1.7–4.2)	3.0 (1.8–4.2)	0.771
LYM, × 10⁹/L	1.5 (1.0–1.9)	1.6 (1.1–1.9)	1.4 (0.9–1.9)	0.345
MONO, × 10⁹/L	0.5 (0.3–0.6)	0.4 (0.3–0.6)	0.5 (0.3–0.6)	0.954
PLT, × 10⁹/L	158.0 (115.8–197.3)	155.0 (116–191)	164 (118–214)	0.378
PT, s	12.0 (11.4–12.9)	12.0 (11.4–12.8)	12.1 (11.6–13.1)	0.141
Child–Pugh score, n (%)				
A	368 (91.5)	257 (91.5)	111 (91.7)	0.294
B	34 (8.5)	24 (8.5)	10 (8.3)	
BCLC, n (%)				
0	39 (9.7)	27 (9.6)	12 (9.9)	0.114
A	287 (71.4)	196 (69.8)	91 (75.2)	
B	69 (17.2)	55 (19.6)	14 (11.6)	
C	7 (1.7)	3 (0.8)	4 (3.3)	
Blood loss (mL)	100 (25–400)	100 (20–350)	100(20–400)	0.633
Surgical approach, n (%)				
Segmental/Lobectomy	322 (80.0)	224 (79.7)	98 (80.9)	0.404
Right hepatectomy	54 (13.4)	41 (14.5)	13 (10.7)	
Left hepatectomy	26 (6.4)	16 (5.6)	10 (8.2)	
Tumor number, n (%)				
Unifocal	338 (84.1)	235 (83.6)	103 (85.1)	0.707
Multifocal	64 (15.9)	46 (16.4)	18 (14.9)	
TTV (cm^3^)	54.5 (15.6–246.3)	71.0 (16.9–216)	63 (14.0–292.9)	0.859
MVI, n (%)				
M0	203 (50.5)	131 (46.7)	72 (59.5)	0.060
M1	145 (36.1)	109 (38.8)	36 (29.5)	
M2	54 (13.4)	41 (14.5)	13 (11.0)	

*IQR* interquartile range, *HBsAg* hepatitis B surface antigen, *AFP* alpha-fetoprotein, *ALB* albumin, *ALT* alanine aminotransferase, *AST* aspartate aminotransferase, *IBIL* indirect bilirubin, *WBC* white blood cell count, *NEU* neutrophil count, *LYM* lymphocyte count, *MONO* monocyte count, *PLT* platelet count, *PT* prothrombin time, *BCLC* Barcelona Clinic Liver Cancer staging system, *TTV* total tumor volume, *MVI* microvascular invasion

### NLR and LMR cut-off values

**Fig. 2:**
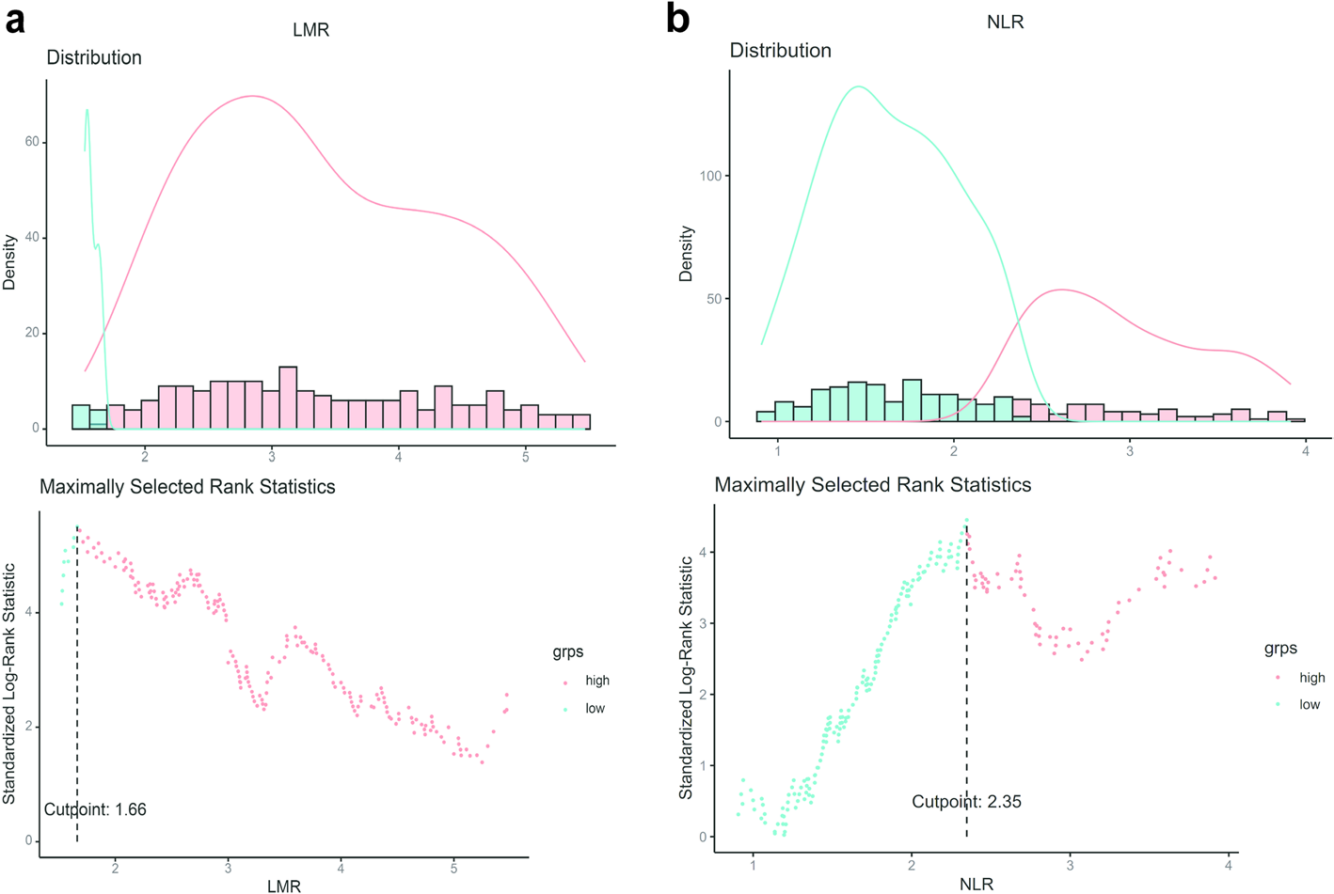
Maximal selection rank statistic was used to determine the optimal cut-off values. Patients (n = 402) were ranked into quartiles based on LMR and NLR values respectively, and the standardized statistic was calculated for each. **a** The largest standardized statistic corresponding to the dashed line in the figure based on LMR values is regarded as the optimal cut—off value. **b** Similarly, the NLR value corresponding to the dashed line in the figure represents the optimal cut—off value

**Fig. 3:**
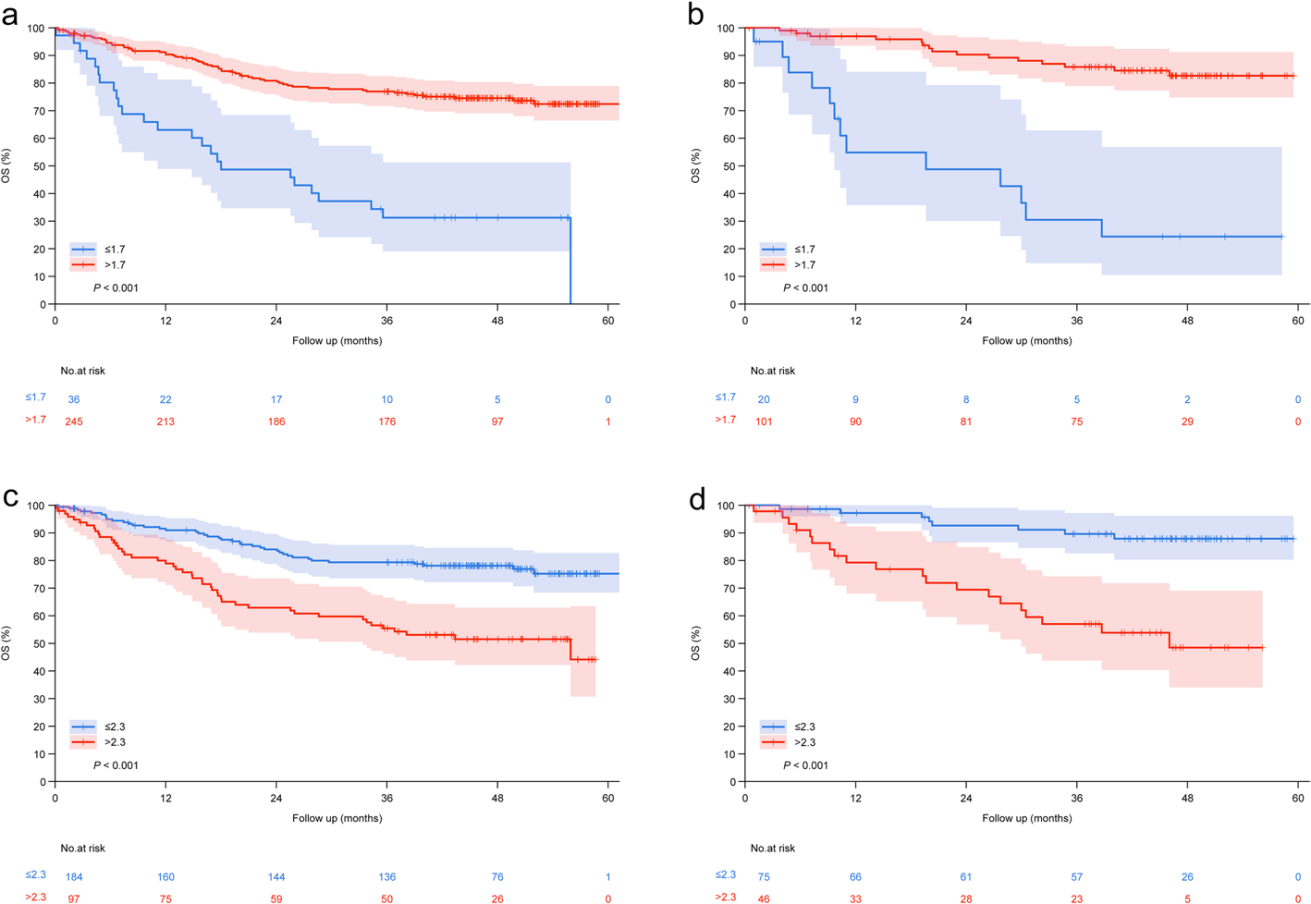
Kaplan–Meier survival curves and log-rank tests were used to validate the associations of LMR and NLR with OS in HCC patients following curative resection. **a** Training cohort LMR analysis. Patients with low LMR (≤ 1.7) exhibited significantly worse OS compared to the high LMR group (HR = 2.16, 95% CI 1.23–3.80, *P* < 0.001). **b** Test cohort LMR validation. The prognostic discrimination remained consistent (HR = 3.21, 95% CI 2.13–4.90, *P* < 0.001). **c** Training cohort NLR evaluation. Patients with high NLR (> 2.3) demonstrated significantly reduced OS compared to the low NLR group (HR = 1.94, 95% CI 1.72–3.68, *P* < 0.001). **d** Test cohort NLR confirmation. The prognostic significance persisted (HR = 4.56, 95% CI 3.24–6.13, *P* < 0.001). Risk tables below the Kaplan–Meier survival curves present the number of surviving patients at different time points

Preoperative complete blood counts were analyzed to calculate NLR and LMR. Maximal selection rank statistic was employed to determine optimal cutoff values for NLR and LMR. The optimal cut-off values determined by maximally selected rank statistics were 1.7 for LMR (lymphocyte-to-monocyte ratio) and 2.3 for NLR (neutrophil-to-lymphocyte ratio), with prognostic stratification validated through Kaplan–Meier survival analysis (Figs. 2a, b, 3a, b, c, d) and demonstrating significant difference between groups (log-rank *P* < 0.001 for all comparisons).

The diagnostic performance analysis revealed that NLR > 2.3 demonstrated a sensitivity of 46.5% (95% CI: 0.36–0.56) and specificity of 73.3% (95% CI 0.66–0.80) for predicting 36-month OS, whereas LMR < 1.7 showed higher sensitivity (80.0%; 95% CI 0.56–0.86) with comparable specificity (72.1%; 95% CI 0.66–0.78) (Supplementary Materials 1 and 2). The Mann–Whitney U test showed no significant difference in cut-offs between this study and published values: NLR 2.3 (Z =  − 0.926, *P* = 0.36) and LMR 1.7 (Z =  − 1.219, *P* = 0.22) (Supplementary Materials 3 and 4).

The NLR—LMR score was defined as follows: 2 for high NLR (> 2.3) and low LMR (≤ 1.7), 1 for either high NLR or low LMR, 0 for neither.

### Risk factors for OS

The following risk factors associated with OS were identified by univariate analysis: (a) surgical approach, (b) TTV and MVI, (c) serum biomarkers including alanine aminotransferase (ALT), aspartate aminotransferase (AST), NLR, LMR, and the composite NLR-LMR inflammatory prognostic score.

The NLR-LMR score showed strong prognostic value in univariate Cox analysis (HR = 5.07, 95% CI 3.01–8.55; *P* < 0.001) for postoperative survival (Supplementary Material 5). Importantly, the composite NLR-LMR score demonstrated superior discriminative ability to either marker alone in the training cohort, with AUC values of 0.626 for NLR and 0.615 for LMR being surpassed by the composite score's AUC of 0.658 (95% CI 0.563–0.666) (Supplementary Material 6). Furthermore, it significantly reduced multicollinearity, with variance inflation factors (VIF) decreasing from 2.30 and 1.97 to 1.06 for the composite score (Supplementary Materials 7 and 8).

**Fig. 4:**
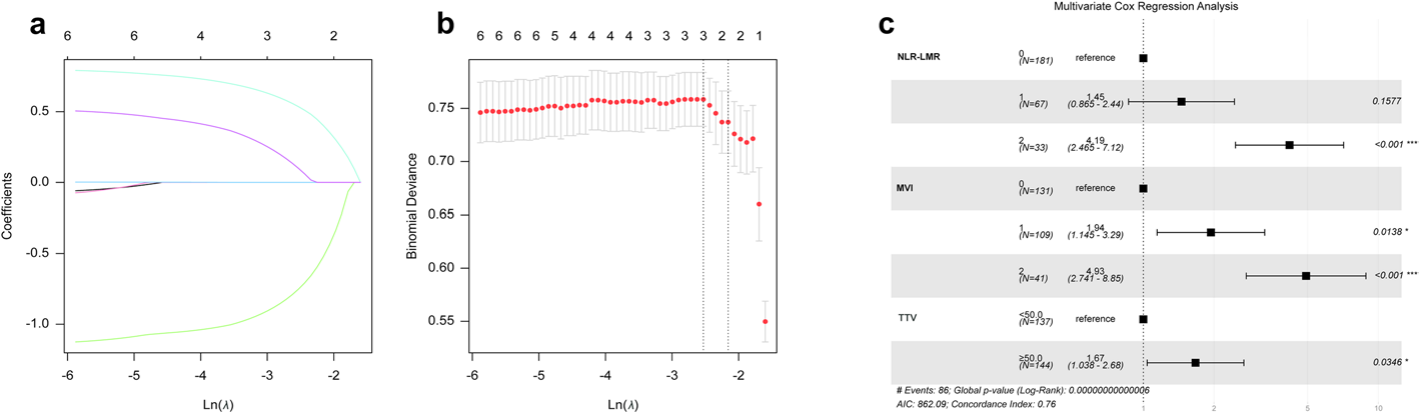
LASSO and multivariate Cox regression analyses were used to identify independent risk factors associated with OS in HCC patients following curative resection. **a** Lasso coefficient trajectories for six variables (MVI, TTV, surgical approach, ALT, AST, NLR-LMR). Variables retaining non-zero coefficients at higher regularization intensities (*λ* ≥ 3) indicate stronger prognostic effects: MVI, TTV, NLR-LMR. **b** Cross-validation curve (y-axis: binomial deviance, lower x-axis: ln(*λ*), upper x-axis: non-zero coefficients) identifies *λ*_min_ = 3 (left dashed line) as the optimal parameter, retaining three predictors with optimal prognostic significance. **c** Multivariable Cox analysis: Tabular data (left) lists HRs (95% CIs), while the forest plot (right) visualizes associations. Horizontal bars (95% CIs) excluding the HR = 1 reference line (vertical dashed) denote statistical significance (*P* < 0.05). Final model integrates MVI (HR = 4.93, 2.74–8.85), NLR-LMR (HR = 4.19, 2.47–7.12), and TTV (HR = 1.67, 1.03–2.68), validated by 1,000 bootstraps (C-index = 0.76)

Given these results, NLR and LMR were excluded from the multivariable analysis to avoid collinearity and optimize model discrimination. LASSO and multivariable Cox proportional hazards analysis revealed that MVI (HR = 4.93, 95% CI 2.74–8.85), NLR-LMR (HR = 4.19, 95% CI 2.47–7.12), and TTV (HR = 1.67, 95% CI 1.04–2.68) were independent risk factors associated with postoperative OS in HCC patients undergoing curative resection (Fig. 4a, b, c).

### Development of a postoperative overall survival nomogram following curative HCC resection

**Fig. 5:**
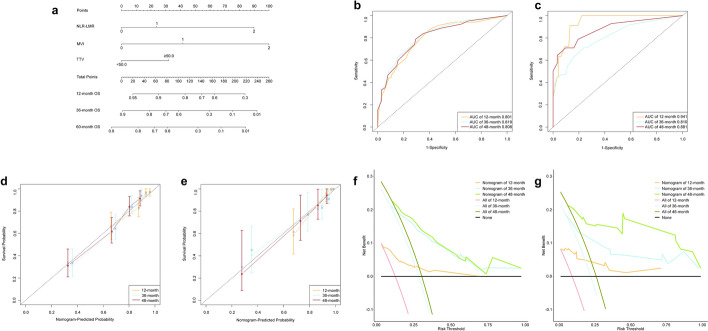
Visualization and validation of nomogram. **a** Nomogram displays three predictors (MVI, TTV, NLR-LMR) with axis lengths proportional to variable weights. The total points are converted into the probabilities of 12-, 36-, and 60-month OS. **b** Training cohort ROC shows area under curves (AUCs) of 0.801 (12-month), 0.819 (36-month), and 0.808 (48-month). **c** Test cohort ROC replicates accuracy (AUCs: 0.941, 0.810, 0.881). **d** Training cohort calibration yields Brier scores (0 = perfect prediction) of 0.095 (12-month), 0.149 (36-month), and 0.158 (48-month). **e** Test cohort calibration maintains precision (Brier Scores: 0.054, 0.120, 0.102). **f** Training cohort DCA shows net benefit over universal treatment ("All") and no-treatment ("None") strategies at threshold probabilities of 0.05–0.72 (12-month), 0.10–0.97 (36-month), and 0.15–0.98 (48-month). **g** Test cohort DCA validates clinical utility (thresholds: 0.02–0.72, 0.05–0.97, 0.06–0.99)

We utilized risk factors identified using multivariable Cox proportional hazards analysis to develop a nomogram for OS in patients who underwent curative HCC resection. Each risk factor was scored separately. NLR-LMR score 0 was considered normal and rated as 0 points, and NLR-LMR score 2 was considered abnormal and rated as 90 points, MVI stage 0 was considered normal and rated as 0 points, and MVI stage 2 was considered abnormal and rated as 100 points, TTV < 50.0 cm^3^ was considered normal and rated as 0 points, and ≥ 50.0 cm^3^ was considered abnormal and rated as 32.5 points. These factors were selected because of their significant association with OS after curative HCC resection. For example, a patient with NLR-LMR score 0, MVI stage 2, and TTV ≥ 50.0 cm^3^ would receive a total score of 132.5 (0 + 100 + 32.5). According to the nomogram, this score corresponds to a predicted 12-month OS probability of 87%, 36-month OS probability of 43%, and 60-month OS probability of 31%. Higher scores indicate lower survival probabilities: elevated NLR-LMR, advanced MVI staging, and larger TTV independently correlate with shorter OS. Total scores (ranging from 0 to 220) correspond to OS probabilities at 12-, 36-, and 60-month (e.g. 0 points = 60-month OS probability of 86%, 220 points = 60-month OS probability of 1%) (Fig. 5a).

### Validation of the accuracy of the nomogram

To further evaluate the model, ROC curves were generated. The area under curve (AUC) values for 12-, 36-, and 60-month OS predictions in the training cohort were 0.801, 0.819, and 0.808, respectively, while corresponding values in the test cohort were 0.941, 0.810, and 0.881, indicating robust discriminative ability (Fig. 5b, c). Calibration curves confirmed high congruence of predicted and observed outcomes across both cohorts. Brier scores for 12-, 36-, and 60-month OS predictions in the training cohort were 0.095, 0.149, and 0.158 (0 = perfect, 1 = worst), compared to 0.054, 0.120, and 0.102 in the test cohort (Fig. 5d, e). And to overcome the limitations of ROC curves in the assessment of clinical utility, DCA was performed. DCA analysis is a mathematical framework that evaluates model effectiveness by quantifying net benefit derived from intervention decisions at varying risk thresholds, facilitating personalized treatment implementation. In DCA analyses, the x-axis represents risk thresholds, and the y-axis denotes net benefit (Fig. 5f, g). In the training cohort, favorable clinical net benefit for 12-, 36-, and 60-month OS predictions was observed at threshold probabilities of 0.05–0.72, 0.10–0.97, and 0.15–0.98, respectively. Corresponding thresholds in the test cohort were 0.02–0.72, 0.05–0.97, and 0.06–0.99. Across both cohorts, DCA analyses demonstrated superior net benefit over the threshold probability range of 0.15–0.70 compared to reference line, confirming broad clinical applicability and utility in most decision-making scenarios.

## Discussion

China carries a disproportionately high global burden of HCC, contributing to the majority of worldwide incidence and mortality cases. Surgical resection remains the primary treatment modality for HCC in China. This study analyzed risk factors associated with post-resection OS in patients undergoing curative HCC resection and developed a nomogram incorporating MVI, NLR-LMR, and TTV to stratify survival outcomes.

Here, we aimed to develop and validate a preoperative inflammatory score-based nomogram for predicting OS in HCC patients following curative resection. Our findings highlight the nomogram's potential as a clinical tool for postoperative risk stratification.

Studies have reported that NLR predicts survival outcomes in patients with distinct malignancies, such as lung, urothelial, and gastric cancers [1, 20]. In primary liver cancer, elevated baseline NLR scores correlate with tumor differentiation and primary TTV, validated across multicenter cohorts [21–23]. Phenotypically, patients with high NLR scores demonstrate more aggressive tumor phenotypes, underscoring NLR as a high-risk biomarker [24]. In surgical contexts, post-resection NLR elevation predicts poorer progression-free survival in HCC patients treated with transcatheter arterial chemoembolization (TACE), further confirming its relevance to HCC outcomes [25–27]. LMR also demonstrated strong diagnostic value in predicting the progression and survival of patients with various malignancies, such as lung and colorectal cancers [28, 29]. Immunologically, emerging evidence links low LMR to programmed death-ligand 1 (PD-L1) expression and poor response to PD-L1 inhibitors, expanding its clinical utility in treatment decision-making [30, 31]. In terms of tumor invasion, inflammatory cells exhibit a close relationship with the tumor microenvironment. Monitoring inflammatory cells can reflect changes in the tumor microenvironment, thereby monitoring the invasion and metastasis of tumor cells. These findings have been preliminarily validated and remain an area of active investigation [32]. In surgical contexts, similar to NLR, post-resection low LMR correlates with shorter OS in HCC patients who underwent radiofrequency ablation (RFA), TACE, or degradable starch microsphere-based chemoembolization (DSM-TACE) [30, 33–35].

Extensive research has well established the prognostic value of NLR and platelet-to-lymphocyte ratio (PLR) as systemic inflammatory markers in HCC, and different prognostic nomogram models to predict OS in HCC patients under various treatment modalities. Multiple studies have demonstrated that the combination of NLR and PLR effectively predicts post-liver transplantation survival [36–43], while integrating NLR with albumin-bilirubin (ALBI) grade improves prognostic accuracy in patients undergoing curative resection [44]. Furthermore, advanced nomogram models incorporating NLR and other inflammatory markers have shown significant predictive capacity for OS across various HCC treatment modalities including curative resection, transarterial chemoembolization (TACE), and microwave ablation (all *P* < 0.05) [45–48]. These models are based on the well-recognized pathophysiological mechanisms: NLR reflects cytokine storm-mediated systemic inflammation, while PLR indicates tumor metastatic potential, together representing comprehensive inflammatory status in HCC patients.

In contrast to these established markers, the prognostic significance of preoperative NLR combined with preoperative LMR in HCC patients undergoing curative resection remains insufficiently investigated. The existing literature presents conflicting evidence: Wang S et al. [49] reported that LMR lacked significant predictive value for OS in HCC patients following microwave ablation (*P* > 0.05). Similarly, Itoh S et al. [30] and Haruki K et al. [50] found neither preoperative NLR nor preoperative LMR reached statistical significance in predicting post-resection OS in HCC patients (*P* > 0.05). Conversely, Wang C et al. [51] demonstrated that NLR and LMR measured at recurrence were significant predictors of post-recurrence survival (PRS) in patients with early HCC recurrence (within 2 years post-hepatectomy, *P* < 0.05). Supporting our findings, Yang T et al. [52] confirmed both preoperative NLR and preoperative LMR as independent prognostic factors for OS in HCC patients after curative resection (*P* < 0.05). These contradictory results highlight the need for further investigation into the combined prognostic value of NLR and LMR.

Our study, through an independent patient cohort compared to previous studies, has further validated the prognostic value of combined NLR and LMR assessment, providing significant complementary evidence to address existing controversies regarding their individual prognostic significance. Moreover, we have established a prognostic nomogram incorporating the composite NLR-LMR scoring system. Using the maximal selection rank statistic, we determined the optimal cut-offs for NLR and LMR, and constructed an NLR-LMR joint scoring system to divide patients into three different prognostic subgroups, with statistically significant differences in survival (*P* < 0.05). ROC analysis revealed superior predictive performance of the combined NLR-LMR score compared to individual markers (AUC: NLR alone 0.626 vs. LMR alone 0.616 vs. combined 0.658).

LASSO and multivariable Cox proportional hazards analysis were used to identify additional factors associated with survival after curative HCC resection. We developed a nomogram for post-resection OS using these variables and evaluated using ROC curves, calibration curves, and DCA. The nomogram exhibited robust discriminative performance, with AUC values of 0.801 (12-month), 0.819 (36-month), and 0.808 (60-month) in the training cohort, and 0.941, 0.810, and 0.881 respectively in the test cohort. Calibration curves demonstrated close agreement between predicted and observed outcomes (Brier scores close to 0 in both cohorts). Clinically applicable across a wide risk threshold range (0.15–0.70), this tool provides reliable post-resection stratification, with potential to guide intensified surveillance (e.g., 3-month imaging) for high-risk patients (defined as those with a nomogram-predicted 60-month OS probability < 60%, corresponding to total points > 82).

Traditional post-resection risk factors, such as TTV and differentiation, are solely pathological in nature. This study incorporates preoperative inflammatory markers reflecting the survival of tumor patients to develop a convenient, accessible, cost-effective, and reliable nomogram for HCC after hepatectomy. The nomogram, which includes the NLR-LMR composite score, provides a reference for post-resection intervention and surveillance decisions, supplementing existing prognostic criteria to enable more efficient and accurate risk stratification. This advancement holds significant implications for prolonging OS in HCC patients following resection.

Notwithstanding its contributions, this study has limitations. First, it is a single-center retrospective investigation. Second, due to the lack of universally accepted NLR and LMR cutoff values, the thresholds used in this analysis were derived from institutional data, which may vary across centers and influence risk stratification outcomes. Third, this study did not include comparative analyses with other established prognostic indicators (e.g., SII, PNI, GPS). These limitations highlight the need for future multicenter prospective studies to further validate these findings.

## Conclusion

The nomogram developed by our team incorporating NLR-LMR scores, enables OS-based risk stratification in HCC patients after hepatectomy, thereby facilitating personalized clinical management.

## Supplementary Information

Additional file 1.

Additional file 2.

Additional file 3.

Additional file 4.

Additional file 5.

Additional file 6.

Additional file 7.

Additional file 8.

## Data Availability

The datasets analyzed during the current study are not publicly available due to privacy restrictions, but are available from the corresponding author upon reasonable request.
