# Supplementary material for: A preoperative inflammatory score-based nomogram predicts overall survival after curative hepatectomy for hepatocellular carcinoma
Source: Discov Oncol. 2025 Aug 31;16:1659. doi: 10.1007/s12672-025-03406-1 (PMC12399463; doi:10.1007/s12672-025-03406-1)
Supplement: Supplementary file 1 — Additional file 1. [file 12672_2025_3406_MOESM1_ESM.pdf]

VassarStats Printable Report:
From an Observed Sample: Estimates of Population Prevalence, Sensitivity, Specificity, Predictive Values, and likelihood Ratios

Fri Jun 06 2025 23:35:01 GMT+0800 (中国标准时间))

Values entered:

	Condition		
	Absent	Present	Totals
Test Positive	48	47	95
Test Negative	132	54	186
Totals	180	101	281

	Estimated Value	95% Confidence Interval	
		Lower Limit	Upper Limit
Prevalence	0.359431	0.303876	0.418896
Sensitivity	0.465347	0.366428	0.566931
Specificity	0.733333	0.661424	0.795078
For any particular test result, the probability that it will be:			
Positive	0.338078	0.283597	0.397066
Negative	0.661922	0.602934	0.716403
For any particular positive test result, the probability that it is:			
True Positive	0.494737	0.391333	0.598571
False Positive	0.505263	0.401429	0.608667
For any particular negative test result, the probability that it is:			
True Negative	0.709677	0.637921	0.772602
False Negative	0.290323	0.227398	0.362079
likelihood Ratios: [C] = conventional [W] = weighted by prevalence			
Positive [C]	1.74505	1.267191	2.403109
Negative [C]	0.729073	0.605388	0.878027
Positive [W]	0.979167	0.73678	1.301294
Negative [W]	0.409091	0.325362	0.514367
