# Supplementary material for: A preoperative inflammatory score-based nomogram predicts overall survival after curative hepatectomy for hepatocellular carcinoma
Source: Discov Oncol. 2025 Aug 31;16:1659. doi: 10.1007/s12672-025-03406-1 (PMC12399463; doi:10.1007/s12672-025-03406-1)
Supplement: Supplementary file 2 — Additional file 2. [file 12672_2025_3406_MOESM2_ESM.pdf]

VassarStats Printable Report:
From an Observed Sample: Estimates of Population Prevalence, Sensitivity, Specificity, Predictive Values, and likelihood Ratios

Fri Jun 06 2025 23:46:25 GMT+0800 (中国标准时间))

Values entered:

	Condition		
	Absent	Present	Totals
Test Positive	68	27	95
Test Negative	176	10	186
Totals	244	37	281

	Estimated Value	95% Confidence Interval	
		Lower Limit	Upper Limit
Prevalence	0.131673	0.095525	0.178195
Sensitivity	0.72973	0.556127	0.856279
Specificity	0.721311	0.659817	0.775698
For any particular test result, the probability that it will be:			
Positive	0.338078	0.283597	0.397066
Negative	0.661922	0.602934	0.716403
For any particular positive test result, the probability that it is:			
True Positive	0.284211	0.198708	0.387436
False Positive	0.715789	0.612564	0.801292
For any particular negative test result, the probability that it is:			
True Negative	0.946237	0.900538	0.972448
False Negative	0.053763	0.027552	0.099462
likelihood Ratios:<br>[C] = conventional<br>[W] = weighted by prevalence			
Positive [C]	2.618442	1.976154	3.469486
Negative [C]	0.374693	0.219983	0.638208
Positive [W]	0.397059	0.281667	0.559723
Negative [W]	0.056818	0.031073	0.103893
