# Supplementary figures and images for: A preoperative inflammatory score-based nomogram predicts overall survival after curative hepatectomy for hepatocellular carcinoma
Source: Discov Oncol. 2025 Aug 31;16:1659. doi: 10.1007/s12672-025-03406-1 (PMC12399463; doi:10.1007/s12672-025-03406-1)

ROC Curve Comparison

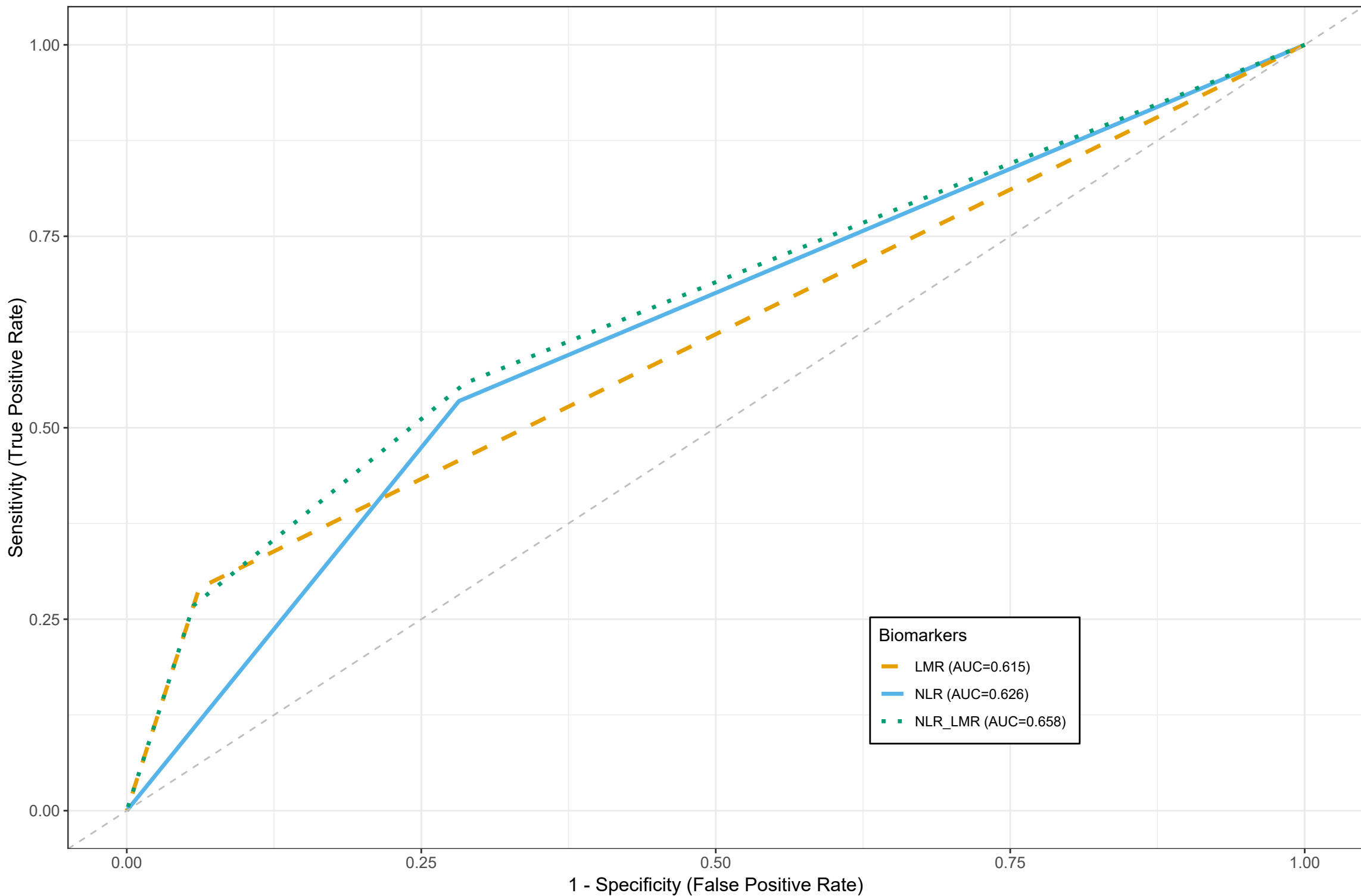

Supplement: Supplementary file 6 — Additional file 6. [file 12672_2025_3406_MOESM6_ESM.pdf]
